# Genetic determinants of Vitamin D deficiency in the Middle Eastern Qatari population: a genome-wide association study

**DOI:** 10.3389/fnut.2023.1242257

**Published:** 2023-09-29

**Authors:** Nagham Nafiz Hendi, Yasser Al-Sarraj, Umm-Kulthum Ismail Umlai, Karsten Suhre, Georges Nemer, Omar Albagha

**Affiliations:** ^1^Division of Biological and Biomedical Sciences, College of Health and Life Sciences, Hamad Bin Khalifa University, Doha, Qatar; ^2^Division of Genomics and Translational Biomedicine, College of Health and Life Sciences, Hamad Bin Khalifa University, Doha, Qatar; ^3^Qatar Genome Program (QGP), Qatar Foundation Research, Development and Innovation, Qatar Foundation (QF), Doha, Qatar; ^4^Bioinformatics Core, Weill Cornell Medicine-Qatar, Doha, Qatar

**Keywords:** Vitamin D deficiency, genome-wide association study, genetic predispositions, polygenic risk score, Middle Eastern

## Abstract

**Introduction:**

Epidemiological studies have consistently revealed that Vitamin D deficiency is most prevalent in Middle Eastern countries. However, research on the impact of genetic loci and polygenic models related to Vitamin D has primarily focused on European populations.

**Methods:**

We conducted the first genome-wide association study to identify genetic determinants of Vitamin D levels in Middle Easterners using a whole genome sequencing approach in 6,047 subjects from the Qatar Biobank (QBB) project. We performed a GWAS meta-analysis, combining the QBB cohort with recent European GWAS data from the UK Biobank (involving 345,923 individuals). Additionally, we evaluated the performance of European-derived polygenic risk scores using UK Biobank data in the QBB cohort.

**Results:**

Our study identified an association between a variant in a known locus for the group-specific component gene (*GC*), specifically rs2298850 (*p*-value = 1.71 × 10^−08^, Beta = −0.1285), and Vitamin D levels. Furthermore, our GWAS meta-analysis identified two novel variants at a known locus on chromosome 11, rs67609747 and rs1945603, that reached the GWAS significance threshold. Notably, we observed a moderately high heritability of Vitamin D, estimated at 18%, compared to Europeans. Despite the lower predictive performance of Vitamin D levels in Qataris compared to Europeans, the European-derived polygenic risk scores exhibited significant links to Vitamin D deficiency risk within the QBB cohort.

**Conclusion:**

This novel study reveals the genetic architecture contributing to Vitamin D deficiency in the Qatari population, emphasizing the genetic heterogeneity across different populations.

## 1. Introduction

Vitamin D is a steroid hormone and nutrient that modulates mineral homeostasis. The level of the circulating Vitamin D metabolite, 25-hydroxy-Vitamin D (25(OH)D), is a biomarker that indicates Vitamin D status. Concentrations of 25(OH)D less than 20 ng/mL (50 nmol/L) are considered the most common nutritional deficiency worldwide (1). Of vital importance, this deficiency can lead to severe clinical manifestations, such as osteoporosis and rickets in children. Environmental and clinical factors, including limited exposure to UVB radiation due to latitude or cultural reasons, obesity, skin pigmentation, advanced age, and genetics, are the leading causes of Vitamin D deficiency worldwide (2). Genetics, in particular, plays a significant role in determining 25(OH)D levels, contributing between 23 and 90% of the variation observed in twin and familial studies (3–5).

Despite significant knowledge about Vitamin D epidemiology, the downstream pathways by which genetic markers affect Vitamin D levels in diverse global populations remain to be fully elucidated (6). European GWAS have identified multiple SNPs linked to genes responsible for Vitamin D transportation (*GC, APOA1*), biosynthesis (*DHCR7*, *NADSYN1*), metabolism (*CYP2R1*, *RRAS2*, *PDE3B*, *CYP24A1*, and *AMDHD1*), and activity (*VDR* and *RXR*) (7–10). Common genetic signatures with minor allele frequency (MAF) greater than 5% can be used to predict individuals at risk of Vitamin D deficiency and guide their personalized therapeutic strategies (11). For example, recent genetic epidemiological evidence recommends a Vitamin D-enriched diet and supplementation for individuals at high risk of multiple sclerosis (12).

Despite the abundance of sunlight in Middle Eastern regions, there is a remarkably high prevalence of Vitamin D deficiency (13, 14), with up to 90% incidence reported in Qatar (15). Previous GWAS have primarily focused on identifying Vitamin D polymorphisms and evaluating the performance of polygenic risk scores in Europeans (7–10). However, no such studies have been conducted on Middle Easterners. Given the high incidence reported in sunny regions, characterizing the genetic architecture underlying Vitamin D pathways in these populations is crucial. We conducted the first GWAS of Vitamin D levels in Middle Eastern individuals using a whole-genome sequencing approach. To validate our results, we combined the QBB GWAS data with a previous large GWAS dataset of 345,923 individuals in a meta-analysis (8). We also evaluated the performance of European-derived polygenic risk scores (PRS) in the QBB cohorts. We assessed the association between genetic markers related to Vitamin D deficiency and phenotype severity for the first time.

## 2. Methods

### 2.1. Study participants

Data used in the present study were obtained from the Qatar Biobank (QBB) dataset. The QBB cohort is the first population-based prospective cohort study that included participants of Qatari nationals or long-term residents (living in Qatar for ≥15 years) and aged 18 years and older. Physical measurements for all participants were collected during the assessment session, and each participant completed a standardized questionnaire reporting lifestyle, diet, and medical history information. In addition, detailed baseline sociodemographic data, phenotypic data, clinical biomarkers, and biochemical tests were collected for the study participants. More details of the QBB project are explained previously (16).

All QBB participants signed informed consent waivers before participation. We submitted a request to access the QGP and QBB data,^1^ which was approved by the QBB IRB (IRB project number, QF-QGP-RES-ACC-00075). The first QBB dataset release (*N* = 6,218 individuals) was used to carry out the current GWAS analysis. A large GWAS published recently using European, African, and South Asian participants were used in performing the meta-analysis and replication analyses (*N* = 363,228 individuals) (8).

### 2.2. Circulating 25(OH)D and dependent covariates

Blood samples were collected, centrifuged for serum separation, and immediately stored at −80°C for all participants. Quantitative evaluations of serum 25(OH)D concentrations were analyzed using a fully automated chemiluminescent immunoassay (CLIA), DiaSorin LIAISON, Germany, in the diagnostic laboratories at Hamad Medical City. Briefly, serum Vitamin D was dissociated from Vitamin D binding protein, and a labeled tracer was added. A washing step was performed to remove any unbound protein before initiating the CLIA reaction. 25(OH)D levels were determined using a photomultiplier. Serum concentration of 25(OH)D were available for 5,885 subjects. Before the statistical analyses, the phenotype was normalized using rank-based inverse standard transformation by R (version 3.4.0). The anthropometric measurements, such as body weight and height, were performed by the Seca 284 stadiometer and balance. Body mass index (BMI) was calculated by dividing the weight (kg) by the square of height (m^2^).

### 2.3. Whole genome sequencing and bioinformatics analysis

Genomic DNA was isolated from whole peripheral blood using an automated QIASymphony SP instrument following the Qiagen MIDI kit protocol’s instructions (Qiagen, Germany). Quantification was performed on the FlexStaion 3 (Molecular Devices, United States) using Quant-iT dsDNA Assay (Invitrogen, United States). Whole genome sequencing was conducted on the Illumina HiSeq X Ten (Illumina, United States) platform with an average coverage of 30x at Sidra Clinical Genomics Facility as previously described (17). Briefly, FastQC (https://www.bioinformatics.babraham.ac.uk/projects/fastqc/; version 0.11.2) were aligned to the human reference genome GRCh37 (hs37d53) using Burrow-Wheeler Aligner (version 7.12; BWA, https://github.com/lh3/bwa/tree/master/bwakit). Variant calling was obtained using HaplotypeCaller provided by Genome Analysis Toolkit (GATK, https://software.broadinstitute.org/gatk/documentation/article?id=3238; version 3.4). Joint calling was conducted on all individual intermediate genomic variant call files (gVCF) to create a joint multi-samples VCF file for all the samples. The process consisted of two steps. We first applied GenomicsDB to combine regions for all samples. We then utilized GenotypeGVCFs using SNP/Indel recalibration to merge all regions. Variants with the PASS filter were only included for downstream analysis following the GATK VQSR filtering steps.

A comprehensive quality control (QC) assessment was performed to minimize population structure and genetic diversity in the QBB data using PLINK (version 2.0) (18). SNPs with genotyping call rate < 90%, the minor allele frequency (MAF) < 1%, or the Hardy–Weinberg equilibrium *p* < 1 × 10^−6^ were excluded. Additionally, we excluded samples with call rate < 95% (*N* = 1), duplicates (*N* = 10), excess heterozygosity (*N* = 8), and gender ambiguity (*N* = 65). We also performed multidimensional scaling (mds) analysis to identify population ancestry outliers with PLINK (18). A set of pruned independent autosomal SNPs (*N* = 62,475) was used to determine the pairwise identity-by-state (IBS) matrix through a window size of 200 SNPs and LD threshold of *r*^2^ = 0.05. Population outliers were considered (*N* = 87) and excluded if they deviated from the mean of the first two mds components by four standard deviation units or more (±4 SD). The genome-wide association analysis was conducted on 7,880,618 genetic variants obtained from 6,047 participants, using the sample with available phenotype data, including 5,927 individuals.

### 2.4. Genome-wide association analysis

Genome-wide association analysis (GWAS) under a variance component-based linear model was performed using GRAMMAR-Gamma (19) within the GenABEL/R package (version 1.8-0) (20) to study the association of each variant and 25(OH)D levels. This method corrects for relatedness and genetic substructure by using the genomic kinship matrix. Considering the mixture of the Qatari population, we performed principal components analysis through PLINK software (18). The first four population principal components (PCs) were used as covariates in the association model to minimize bias from population stratification. Further, we adjusted the regression model for age (years) and gender. The sample collection for this study was conducted during a similar sunny season in Qatar, and therefore, we did not consider seasonality as a confounding factor in our analysis. Genome-wide significance cutoff was defined as *p <* 5 × 10^−8^ and the nominal significance level as *p* < 0.05 (21). The quantile-quantile (Q-Q) plot, Manhattan plot, and genomic inflation factor were generated by R (version 3.4.0). Heritability was identified as part of the polygenic risk model in GenABEL to estimate the degree of variation in the 25(OH)D levels due to inter-individual genetic variation in a population (22).

To assess pairwise linkage disequilibrium (LD) between significant SNPs, we conducted LD clumping analysis using PLINK software (version 1.9). We utilized GenABEL summary statistics obtained from the GWAS analysis of the QBB cohort with an *r*^2^ threshold of 0.2, identifying SNPs in strong LD with each other. To visualize the LD patterns, we employed LocusZoom software (23) to generate LD plots based on the GenABEL summary statistics, highlighting clusters of SNPs in high LD.

### 2.5. Meta-analysis

We combined the results of the QBB GWAS and a recent large GWAS (GCST90019526) from the United Kingdom Biobank by the Sinnott-Armstrong et al. (8) (*N* = 363,228 individuals) to derive a combined meta-analysis for the suggestively associated loci. The United Kingdom Biobank GWAS models were characterized with the same phenotype and methods following the correction of relevant covariates, including age, sex, and genotype PCs (8). Summary statistics for the United Kingdom Biobank study (8) were obtained from the NHGRI-EBI GWAS Catalog (24) taken on December 02, 2022. We confirmed matching A1 and A2 alleles with the alternate and reference alleles in the United Kingdom Biobank study. We also canonicalized the QBB association statistics as the reported effect size based on the alternate allele in the reference genome. Notably, 25(OH)D measurements were inversely normalized using rank-based transformation in both GWASs. This technique involves ranking the values in ascending order and then transforming the ranks to a normal distribution using the inverse of the cumulative distribution function. It is designed to adjust for outliers and skewness in the non-normally distributed traits (25). PLINK (version 1.9) was utilized to perform an inverse-variance weighted meta-analysis and estimate heterogeneity of effects analysis (18).

### 2.6. Validation of previous association with 25(OH)D

We compared the QBB findings to those of the United Kingdom Biobank GWAS study on Vitamin D from the United Kingdom Biobank project (8) to evaluate the extent of replication and correlation of effect size and allele frequency for common signals. To test the possibility that SNPs observed in our meta-analysis were previously reported in the United Kingdom biobank at *p* < 5.0 × 10^−8^, each SNP was checked in the GWAS catalog (EFO_0004631) of Vitamin D measurement trait from the November 2022 released data that was accessed on December 02, 2022 (24). We first examined the locus associated with Vitamin D levels in QBB and United Kingdom biobank driven by the same variants. We then determined markers within a 250-kb upstream and downstream region of the GWAS catalog signals to identify significant variants associated with Vitamin D.

### 2.7. Polygenic scores analysis

We tested the performance of European-derived PRS on the QBB cohort to estimate genetic liability to Vitamin D using PLINK (version 1.9) (18). Polygenic scores consist of combined SNPs by the sum of risk alleles, which are weighted by corresponding effect sizes predicted by GWAS results. We used polygenic risk scores derived from one of the largest Vitamin D GWAS carried out in European populations by Sinnott-Armstrong et al. (8) (PGS000702: *n* = 255,256 individuals and 8,012 variants). The scoring files of the study were obtained via the Polygenic Score Catalog^2^ (26) accessed on December 09, 2022. Pearson’s correlation (*R*) between the inverse normalized Vitamin D levels, and European-derived PRS was computed with adjustment of age, gender, and first four PCs using the R software to evaluate the performance of the models on the Qatari population. We also used the area under the receiver operating characteristic (ROC) curve, also known as the “area under curve” (AUC). The AUC ranges from 0.5 (no distinction) to 1 (complete distinction), indicating the effectiveness of the derived PRSs in identifying those with Vitamin D deficiency, defined as serum 25(OH)D levels below 20 ng/mL and Vitamin D insufficiency when 25(OH)D levels between 21 and 30 ng/mL.

### 2.8. SNP annotation and functional analysis

The identified Vitamin D associations from the GWAS and meta-analysis were annotated using the Ensembl Variant Effect Predictor release 108 (VEP, https://grch37.ensembl.org/index.html) (27). We used the Genome Aggregation Database (gnomAD, https://gnomad.broadinstitute.org) and Allele Frequency Aggregator (ALFA, www.ncbi.nlm.nih.gov/snp/docs/gsr/alfa/) to compare the frequencies of the identified Vitamin D variants with those in the global populations.

## 3. Results

### 3.1. Study description

The present study used the whole genome sequence data of Qatari participants. The average (± SD) age of QBB participants at the time of study enrollment was 40 (±12.8) years, with an interquartile range of 18–88 years. Among the participants who successfully passed quality control procedures, 56.3% were female (*n* = 3,318). Remarkably, we reported that approximately 50% of the participants had Vitamin D baseline levels below 20 ng/mL. Statistically significant associations were observed between 25(OH)D and both age (Pearson’s coefficient of correlation (R) = 0.26, *p* value = 2.2 × 10^−16^) and gender (*p* value = 4.75×10^−9^). The mean BMI (in kg/m^2^) was approximately similar between both genders, 29.38 (± 6.05), with no significant link to serum 25(OH)D levels. Detailed characteristics of the study participants and phenotype assessment are provided in Tables 1, 2.

**Table 1:** Baseline characteristics of the QBB study population.

Participant characteristic	Male	Female	Total
Age (year)	40.1 (±12.4)	40.3 (±13.0)	40.18 (±12.7)
BMI (kg/m^2^)	28.8 (±5.52)	29.9 (±6.53)	29.41 (±6.14)
Serum 25(OH)D levels (ng/mL)	18.2 (±10.5)	19.9 (±11.3)	19.18 (±11.0)
Sample size	2,588 (43.7)	3,339 (56.3)	5,927

Descriptive statistics are expressed as average (± SD) or number (%) for participants of QBB. BMI, Body mass index; 25(OH)D, 25-hydroxyVitamin D.

**Table 2:** Association between serum 25(OH)D and covariates in the QBB cohorts.

Participant characteristic	*R*	95% CI	*p* value
Age (year)	0.26	0.24–0.28	**2.2 × 10** ^**−16**^
BMI (kg/m^2^)	−0.0095	−0.029-0.023	0.4648
Gender	N/A	1.113–2.232	**4.75×10** ^**−9**^

The statistical analyses of the relationship between 25(OH)D and age, BMI, and gender covariates were conducted using Pearson’s correlation and the Welch’s Two Sample Student’s *t*-test. These analyses were performed on the subset of participants from the QBB study who passed quality control criteria and had available phenotype data, totalling 5,927 individuals. A level of value of *p* < 0.05 was considered significant and denoted in bold. BMI, Body mass index; CI, Confidence interval; and R, Pearson’s Rank coefficient.

### 3.2. Genome-wide association study on 25(OH)D

**Figure 1:**
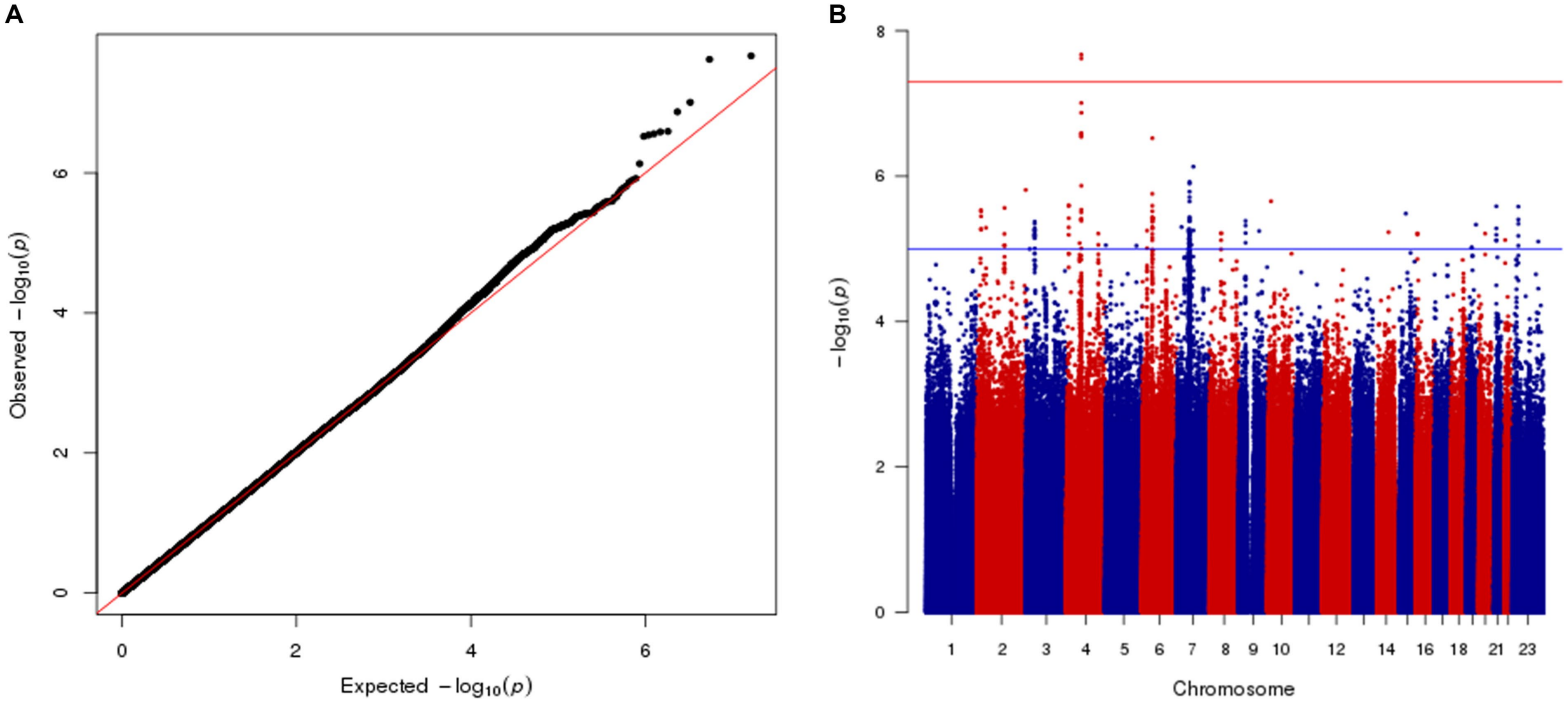
Manhattan and Q-Q Plots illustrating Genome-Wide Association Results for Serum 25(OH)D Levels. **(A)** Manhattan plot for the GWAS performed using linear mixed models correcting for age, gender, the first four principal components, and relatedness. Chromosomal positions of genetic variants (*N* = 7,880,618) plotted against the –log10 *p* value. Genome-wide significance threshold (value of *p* < 5 × 10^−8^) is presented as a horizontal red line. **(B)** Q-Q plot shows the observed –log10 *p* values and the expected –log10 *p* values.

We conducted a genome-wide association study to identify genetic architecture and putative causal genes for Vitamin D in the Middle Eastern Qatari population. The association with circulating 25(OH)D was examined in 6,045 participants who passed quality control (QC). We restricted our examination to common and low-frequency risk alleles (MAF > 1%; *N* = 7,880,618) using linear mixed models correcting for age, sex, PCs, and relatedness (full details in the section “Methods”). Quantile–quantile (Q-Q) and Manhattan plots of genetic associations for circulating 25(OH)D concentrations are shown in Figure 1. The genomic inflation factor of the QBB GWAS did not reveal evidence for widespread inflation [λ_GC_ = 1.01, standard error (SE) = 2.36 × 10^−07^], suggesting no substantial effects of population stratification or cryptic relatedness as revealed in the Q-Q plot (Figure 1B). The Manhattan plot of the GWAS showed a single genome-wide significant signal on chromosome 4q12 as a potential risk locus for 25(OH)D levels in the QBB cohort (Figure 1A).

**Figure 2:**
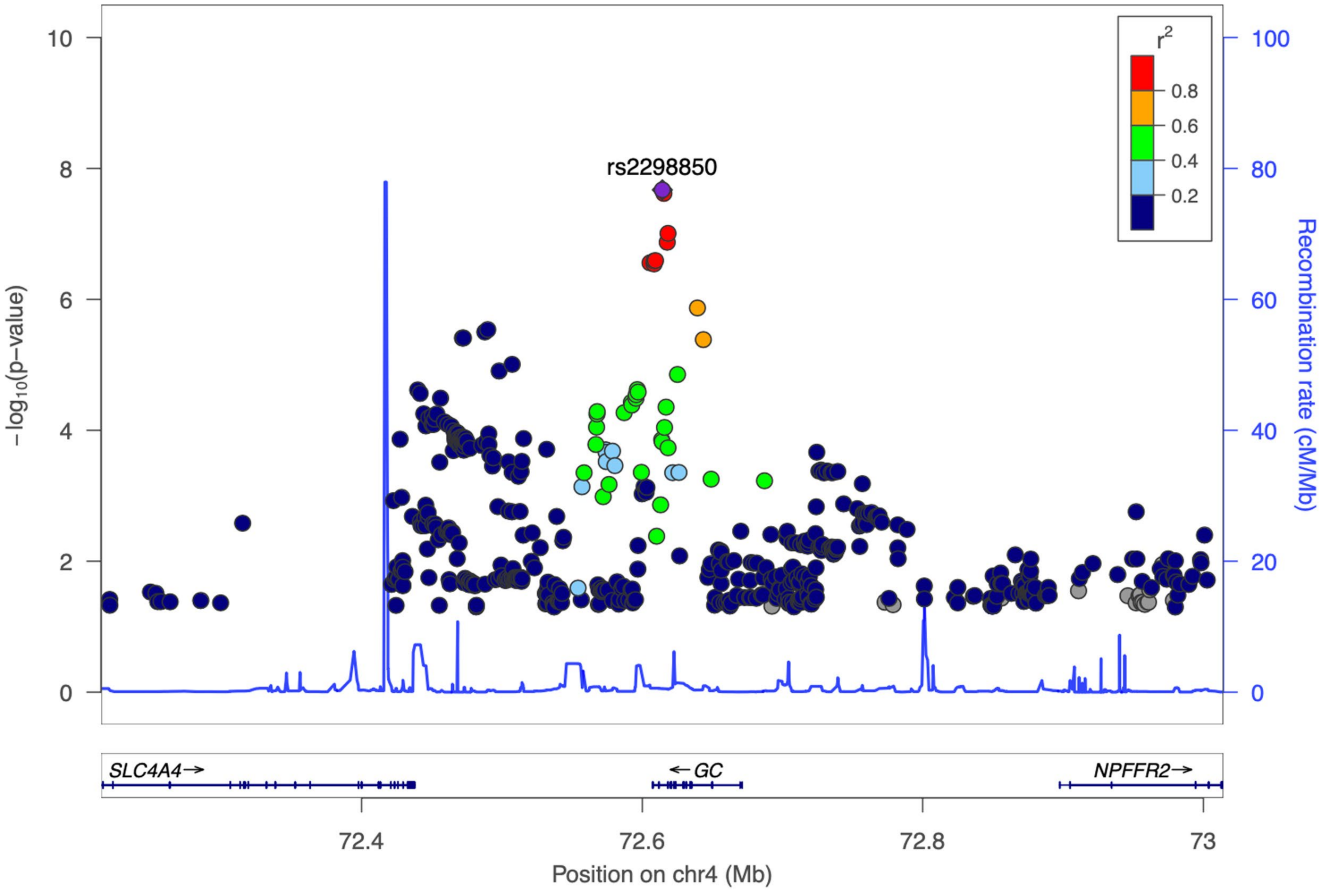
Linkage disequilibrium (LD) plot for the top-associated locus. LocusZoom plots of the leading maker of Vitamin D, rs2298850, on chromosome 4 (in purple diamonds). The left vertical axis of the Manhattan plot represents *p* values as a logarithmic scale, while the right vertical axis shows recombination rates as a blue line, with chromosomal positions being indicated on the horizontal axis. Bottom panel presents gene names and locations. Arrows are utilized to annotate genes within the region, while linkage disequilibrium relationships of each SNP with the lead SNP are depicted in color-coded *r*^2^ values.

We identified three genome-wide significant SNPs at the 4q12 locus (chromosome 4: 72,607,410-72,671,237) with a *p* value of less than 5.0 × 10^−8^ (Table 3). The top-hit markers were located within the region harboring the group-specific component gene (*GC*), which encodes a Vitamin D binding protein (VDBP). Of them, the top-associated variant was rs2298850, located in intron 11 of *GC*, which showed the most significant association with 25(OH)D at a *p* value of 1.71× 10^−08^. The other two SNPs in the *GC* were rs11723621 (*p* value = 1.93 × 10^−08^), followed by rs4588 (*p* value = 8.06 × 10^−08^; Table 3). In our study, we observed that SNPs on chromosome 4 exhibited a high level of LD (Figure 2). This finding suggests that these SNPs are closely correlated, potentially indicating a shared genetic signal or a region of interest. Polymorphisms suggestively linked to 25(OH)D concentrations with a *p* value of less than 5 × 10^−05^ are presented in Supplementary Table 1. We further characterized the GWAS SNPs attribution to 25(OH)D variation (SNP-heritability, *h^2^*) in the Qatari population. The heritability of 25(OH)D using all filtered SNPs was estimated as 18%.

**Table 3:** Genomic variants identified in genome-wide analysis for 25(OH)D levels.

SNP	Gene	HGVS ID	CHR	Position	A1	A2	Beta (SE)	*p* value^*^	MAF (A1)
rs2298850	*GC*	NC_000004.12:g. 71748550G > C	4	72,614,267	C	G	−0.1285 (0.023)	1.71E−8	17.72%
rs11723621	*GC*	NC_000004.12:g. 71749645A > G	4	72,615,362	G	A	−0.1257 (0.022)	1.93E−8	18.52%
rs4588	*GC*	NC_000004.12:g. 71752606G > A	4	72,618,323	T	G	−0.1188 (0.022)	8.06E−8	19.24%

*p value of GWAS analysis using linear mixed models adjusting for age, sex, principal population components, and relatedness was indicated by *p* value ≤ 5.0 × 10^−8^. The analysis is performed using GRCh37/hg19 genome reference. The effect size (Beta) and minor allele frequency (MAF) are reported for allele (A1). SNP, Single nucleotide polymorphism; CHR, Chromosome; A1, Reference allele; A2, Alternative allele; MAF, Minor allele frequency; SE, The standard error for Beta; and GC, Group-specific component. Mapped Genes from ANNOVAR.

### 3.3. Evaluating replication of known loci

We evaluated the extent of replication by comparing our findings to prior published work on Vitamin D from the United Kingdom Biobank (GCST90019526) (8). We chose this study as it is the largest and most comprehensive GWAS on Vitamin D. Furthermore, the UK Biobank study normalized the Vitamin D levels similar to our analysis using rank-based inverse normalization (8). The use of this method facilitated the comparison of effect sizes for identified loci between the two studies. Most of the loci that reached the genome-wide association threshold in the United Kingdom Biobank (8) were replicated in the QBB cohort, around 87% (Supplementary Table 2). For example, the *GC* rs2282679 showed a marginally significant association in the QBB cohort (*p* value = 2.61 × 10^−07^) but had a significant association in the United Kingdom Biobank cohort (*p* value = 1.0 × 10^−1,268^).

**Figure 3:**
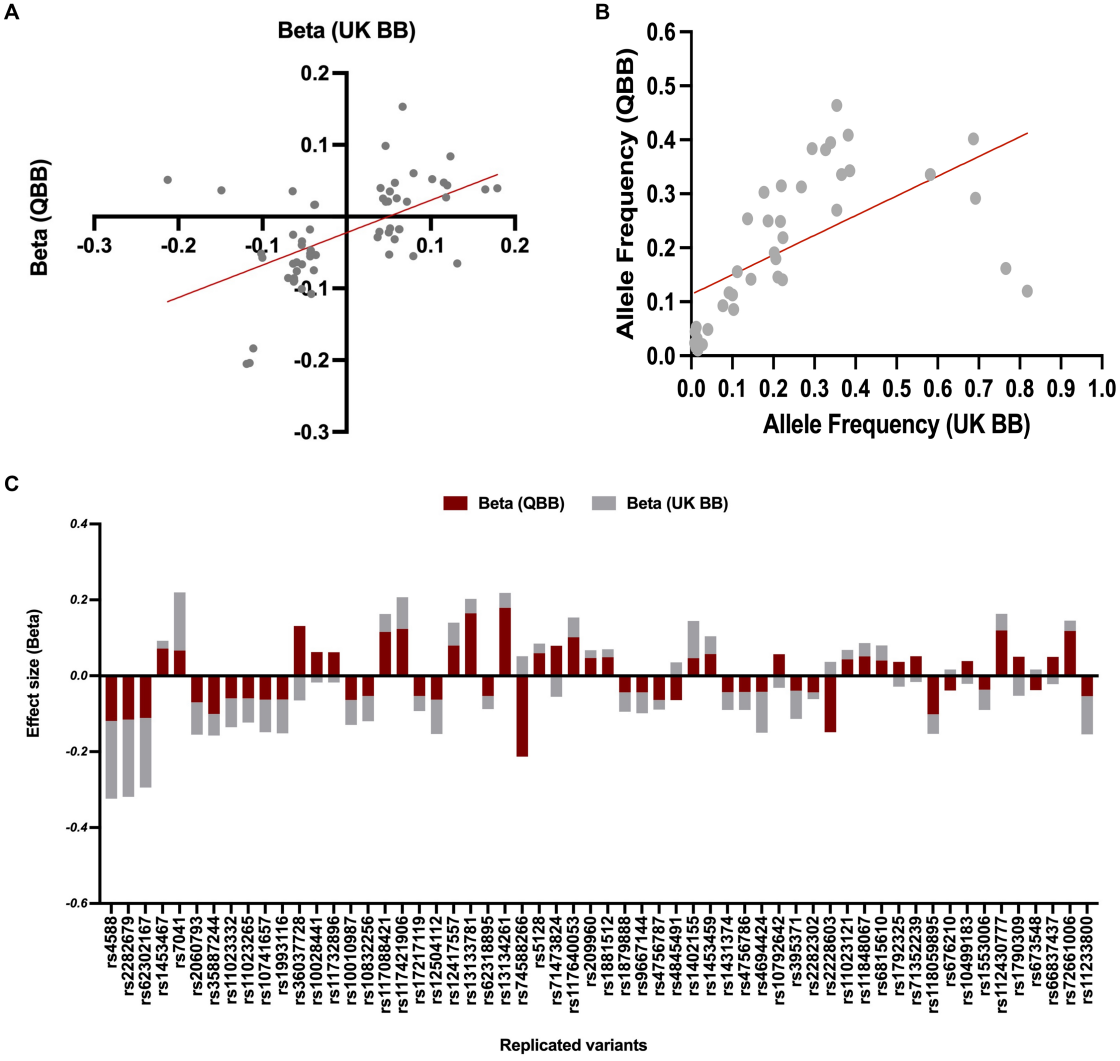
Comparison of allele frequency and effect size for common variants between QBB and United Kingdom Biobank. **(A)** Risk allele frequency correlation for overlapped variants in QBB and European study (*r* = 0.60). **(B)** Effect weight correlation for overlapped variants in QBB and European study (*r* = 0.80). The best-fit line from linear regression analysis is presented as a red line. **(C)** The effect weight (Beta) of SNPs shows replication after correction in the Qatari population (QBB, red bars) compared to the European population (United Kingdom Biobank, gray bars).

Our association analyses of effect directions and effect size for the replicated SNPs (*n* = 58 variants) showed a consistent directionality (Figure 3C), with slightly smaller effect sizes than those reported in the United Kingdom Biobank study (*n* = 43, *R* = 0.8, regression slope = 0.79, 95% CI = 0.63–0.96, value of *p* < 0.0001, Figure 3B). The remaining SNPs showed a reverse association direction compared to QBB, possibly due to underlying differences in study design, population characteristics, or environmental factors. In the United Kingdom Biobank study, the allele frequencies were available for only 40 of the replicated variants, which displayed a Pearson’s coefficient (R) of 0.6 with a value of *p* < 0.0001 (as seen in Figure 3A).

The frequencies of the most significant Vitamin D variants in the QBB cohort were compared with control populations from the gnomAD and ALFA browsers. The frequency of rs2298850 was similar, but rs11723621 and rs4588 were lower in the Qatari population compared to the European population in gnomAD and ALFA (Table 4). In the European data, we identified 43 matching variants with consistent effect sizes for the Vitamin D trait.

**Table 4:** Prevalence of the significant Vitamin D-associated alleles identified in the QBB GWAS.

Populations	Frequency for rs2298850	Frequency for rs11723621	Frequency for rs4588
QBB-Qatari population	0.1772	0.1852	0.1924
European population of ALFA	0.17619	0.28818	0.281206
Controls of gnomAD populations			
European	0.25780	0.27532	0.28121
East Asian	0.2603	0.2593	0.2714
African/African American	0.07066	0.08572	0.09921
All populations	0.1995	0.2189	0.25

gnomAD, Genome aggregation database; ALFA, Allele frequency aggregator.

### 3.4. GWAS meta-analysis for Vitamin D

To detect potential novel variants that have a genome-wide significant association in the QBB GWAS, we combined the QBB GWAS data with a comprehensive European GWAS by Sinnott-Armstrong et al. (8) of similar phenotype (*N* = 363,228 individuals). Details of the replication United Kingdom Biobank study are described previously (8). We identified a total of 35 variants with genome-wide significance in known loci related to Vitamin D. The top-hit variants were rs13361160 in *CYP2R1* (cytochrome P450 2R1; 11:14910234 A < G; Beta = 0.0889, *p* value = 6.38 × 10^−282^), rs12504112 (4:72718873 T > C; Beta = −0.09, *p* value = 8.78 × 10^−189^), followed by rs10832256 (11:14442875 G > A; Beta = −0.07, *p* value = 4.12 × 10^−146^).

Among these associated variants, two genomic markers were below the genome significance threshold in the United Kingdom Biobank study, and reached the genome-wide association threshold upon incorporating data from the QBB cohort, namely rs67609747 (11:15125750 T > C; Beta = −0.02, *p* value = 1.18 × 10^−08^) and rs1945603 (11: 15275158 G > A; Beta = 0.02, *p* value = 2.56 × 10^−08^). Interestingly, these two SNPs on chromosome 11 did not show significant evidence of LD, suggesting their independent genetic signals or association with distinct regions.

Interestingly, the minor allele frequency of rs67609747 was higher, while rs1945603 exhibited almost comparable frequencies in the Qatari population compared to the European population in gnomAD and ALFA datasets (Supplementary Table 3). Our meta-analysis results confirmed the replication of several variants reported similarly in the GWAS Catalog (*n* = 18 variants, Supplementary Table 4), as well as variants located in the same loci of known variants (*n* = 17 variants). Summary of meta-analysis results for the United Kingdom Biobank and QBB cohorts is presented in Supplementary Table 5.

### 3.5. Polygenic risk score estimation

**Figure 4:**
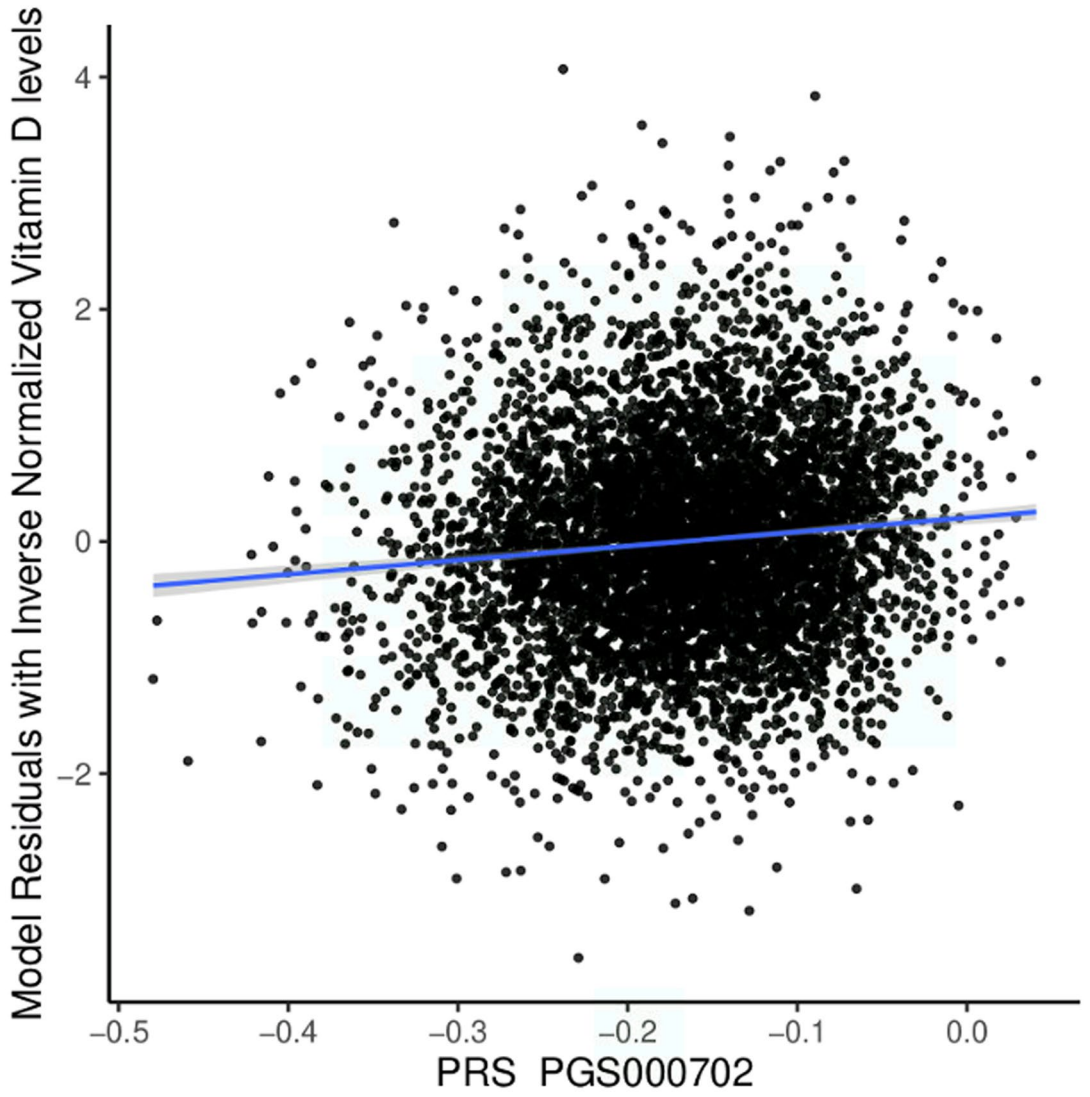
Performance of the European-derived PRS in the Qatari Population. Linear regression of inverse-normalized baseline Vitamin D levels and weighted polygenic risk scores (PRS) derived from a large European dataset (PGS000702: *R* = 0.098, *p* value = 4.60 × 10^−14^). The blue line represents the best fit of linear regression analysis.

We tested the performance of European-derived PRS represented by panel PGS000702 (8) in the QBB cohort against 5,885 individuals with available Vitamin D measurement data, consisting of 3,318 females and 2,567 males. Of the 8,012 variants in panel PGS000702, 6,326 were deemed valid predictors. The scoring process omitted 1,624 variants, with 1,620 being disregarded due to a discrepancy in the variant identifier and four being disregarded due to an allele code mismatch. The performance of the European-derived PRS on the Qatari population for Vitamin D is illustrated in Figure 4.

**Figure 5:**
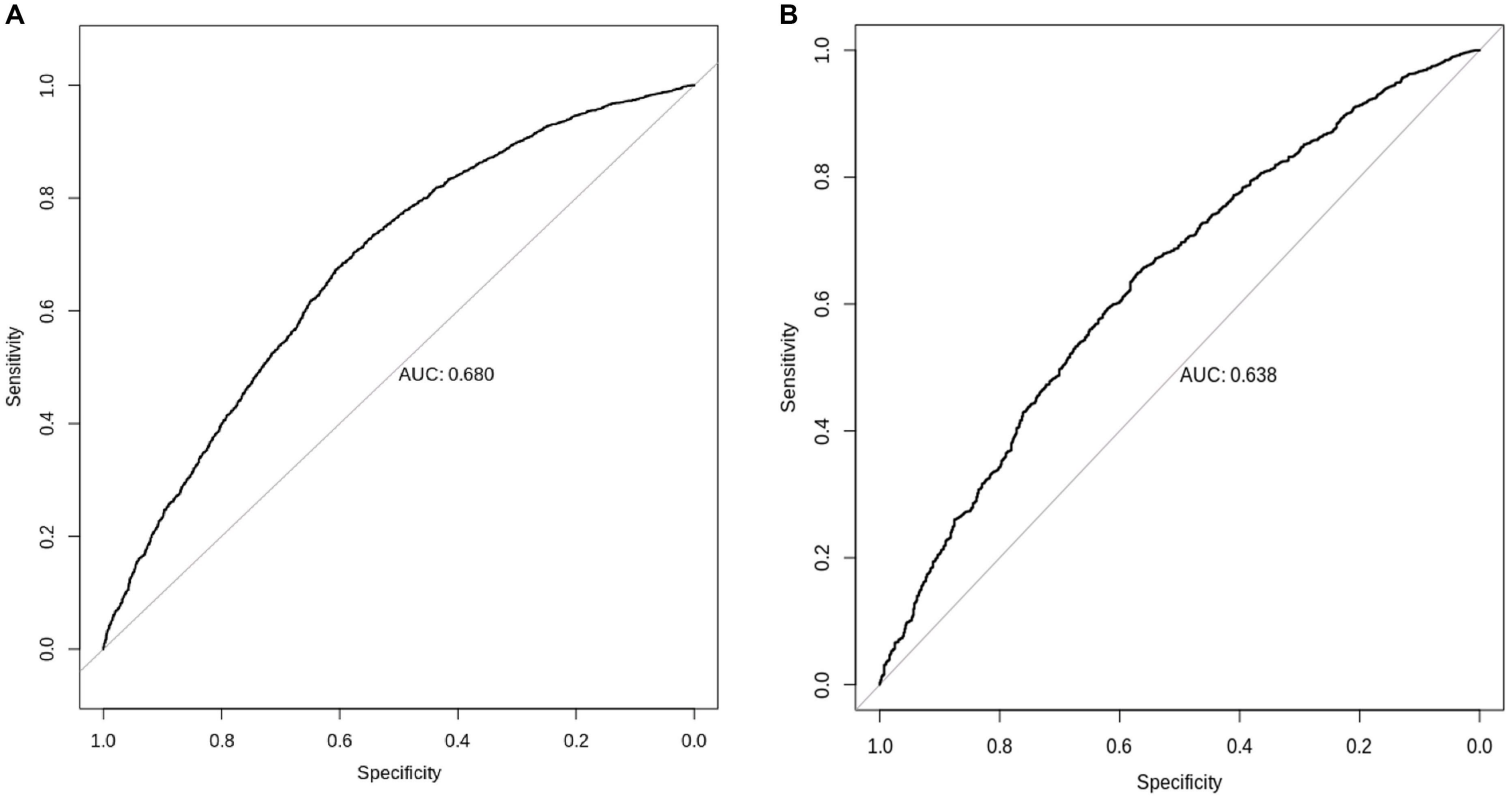
Prediction of Vitamin D status using European-derived PRS in the Qatari Population. Receiver Operating Characteristic (ROC) curve of the European-derived PRS on QBB cohort for the prediction of **(A)** Vitamin D deficiency [25(OH)D < 20 ng/mL], and **(B)** Vitamin D insufficiency and deficiency [25(OH)D < 30 ng/mL]. Area under the ROC curve (AUC) is reported in the image.

We found that European-derived PRS have a lower predictive performance on the QBB cohort (*R* = 0.098, 95% CI = 0.073–0.124, *p* value = 4.60 × 10^−14^) compared to the previously reported *R* values of 0.46 in the European study by the Sinnott-Armstrong et al. (8). Vitamin D PRS was significantly associated with the risk of Vitamin D deficiency with an AUC of 0.680 (*p* value of 4.71 × 10^−9^, Odds Ratio = 0.0935, 95% CI = 0.0420–0.2071; Figure 5A). The risk of Vitamin D insufficiency and deficiency was efficiently predicted with an AUC of 0.6385 (*p* value = 2.28 × 10^−5^, Odds Ratio = 0.0832, 95% CI = 0.0259–0.2646; Figure 5B).

## 4. Discussion

This study presents the first genome-wide association analysis of Vitamin D deficiency in a large cohort of Middle Eastern individuals, consisting of around 6,200 participants. Previous studies have estimated the heritability of 25(OH)D in Europeans to range from 7.5 to 16% (7, 10). However, it is crucial to consider that these estimates can vary based on the population, methods used, and environmental factors affecting 25(OH)D levels. The extent of heritability for Vitamin D in Middle Eastern populations has not yet been established. In this study, we examined the SNP-based heritability of 25(OH)D in the QBB cohort and found it to be slightly higher than that estimated in the United Kingdom Biobank participants, at approximately 18%. This difference can be attributed to various factors, such as geographical location, cultural restrictions, and population-specific genetic architecture (28). Therefore, our study is essential in uncovering genomic markers of Vitamin D in Middle Eastern populations. This finding can potentially aid in the development of targeted interventions for Vitamin D deficiency in this population.

The findings of the GWAS analysis in the Qatari population identified three SNPs linked to Vitamin D on chromosome 4. These markers were located in intron 11 of the *GC* gene, which encodes VDBP, an essential member of the albumin family that synthesizes in the liver and transports Vitamin D and its metabolites (29). The LD analysis results revealed a significant correlation between these three SNPs, suggesting that they may be inherited together as a haplotype.

Importantly, the top-associated marker, rs2298850, did not reach the commonly accepted genome-wide association threshold (*p* value less than 5e−8) in prior GWAS analyses. Nevertheless, we found a weak association between rs2298850 and Vitamin D levels in two genetic studies conducted on pregnant women in China at a *p* value of 0.047 and 0.0009 (30, 31). These studies suggest that rs2298850 may be involved in Vitamin D metabolism in specific populations. However, further research is required to validate this association and explore the underlying mechanisms.

The lack of new loci discoveries reinforces the established understanding of known genes and their interactions in the Vitamin D pathway. Furthermore, candidate gene studies have also shown strong associations between SNPs in the *GC* gene and 25(OH)D concentrations in Middle Easterners (32, 33). While all studies converge on the role of the *GC* gene in Vitamin D deficiency, further investigation of Vitamin D’s genomic background and biological pathways is necessary to improve its clinical management and precision medicine applications.

To increase the statistical power of our findings, we combined data from a large European GWAS (8) with the QBB observations. It is noteworthy that individual alleles may have different genetic backgrounds across populations due to the significant variation in allele frequency and effect size among lineages. Therefore, we analyzed the replication and correlation of identified variants with the United Kingdom Biobank data to examine the consistency of our observations. Our meta-analysis identified 35 SNPs in known loci associated with Vitamin D levels. These SNPs include rs13361160 in *CYP2R1*, which codes a key enzyme in the Vitamin D metabolism pathway (34), and rs10832256 near the *SPON1* (Spondin 1) gene, previously implicated in regulating Vitamin D metabolism (7, 9).

Two new genetic variations in a known locus, namely rs67609747 and rs1945603, have been identified as reaching the genome-wide association threshold through the combination of the United Kingdom Biobank (8) and the QBB cohorts, with no evidence of LD. Both variants are located in an intergenic region of the same locus on chromosome 11 (11:15,125,650–15,275,258), downstream of the calcitonin-related polypeptide beta (*CALCB*) gene. Previous GWAS have established a strong association between *CALCB* and Vitamin D (7–10, 35) and its involvement in various diseases, including diverticular disease (36, 37). This gene plays a regulatory role in the calcium-regulating hormone calcitonin (38). The detected signals in our analysis are most likely due to better coverage of whole genome sequencing and slightly higher allele frequencies in the QBB data compared to the published GWAS, which was based on SNP arrays followed by imputation.

The United Kingdom Biobank cohort is extensive and comprehensive, offering the potential for driving PRS on the QBB population from the European populations. Nevertheless, the predictive performance of European-derived PRS was lower in the Qatari people, which may be attributed to several factors, including the number of variants included, GWAS sample size, the allele frequency of causal variants, and different variant weights among populations. The PRS model for Vitamin D was able to predict deficiency with slightly improved accuracy but not statistically significant when compared to predicting both deficiency and insufficiency. This finding highlights the necessity of a more comprehensive GWAS specific to Middle Eastern people to enhance the accuracy of PRS predictions.

In conclusion, the QBB GWAS has identified for the first time the primary genetic determinant of Vitamin D predisposition in Middle Eastern individuals as a polymorphism in the *GC* gene. Our analysis confirmed previous findings of shared genetic factors among diverse ethnic groups and revealed consistent patterns in the effect size and allele frequency of common variants. The combined analysis of Middle Eastern and United Kingdom Biobank data has led to the identification of two leading genomic markers linked to Vitamin D and the replication of many previously known loci. The poor performance of the European-derived PRS when applied to Middle Eastern individuals underscores the importance of a more comprehensive investigation of Vitamin D genomic variations in non-European populations. The findings provide a valuable understanding of the underlying mechanisms of Vitamin D and the relationship between an individual’s 25(OH)D status and related health issues across Middle Eastern populations.

## Data availability statement

The data used in this study are subject to certain licenses and restrictions. The raw whole genome sequence data from Qatar Biobank cannot be deposited into public databases due to data privacy laws. However, access to the QBB/QGP phenotype and whole genome sequence data can be obtained through an ISO-certified protocol. This involves submitting a project request at https://www.qatarbiobank.org.qa/research/how-apply, which must be approved by the Institutional Review Board of the QBB. If you wish to access these datasets, please visit https://www.qatarbiobank.org.qa/research/how-apply for more information.

## Ethics statement

The studies involving humans were approved by the QBB IRB (IRB project number, QF-QGP-RES-ACC-00075). The studies were conducted in accordance with the local legislation and institutional requirements. The participants provided their written informed consent to participate in this study.

## Author contributions

NH, OA, and GN conceived the idea and designed the model. NH, YA-S, U-KI, and KS developed the codes for analysis of the meta-analyses and polygenic risk score. NH did the data analysis and interpretation of results as well as the write-up of the first draft. All authors contributed to the article and approved the submitted version.

## Footnotes

^1^ https://www.qatarbiobank.org.qa/research/how-to-apply

^2^ https://www.PGSCatalog.org
